# Asymmetry in amyotrophic lateral sclerosis: Clinical, neuroimaging and histological observations

**DOI:** 10.1093/brain/awaf121

**Published:** 2025-04-04

**Authors:** Katie Yoganathan, Thanuja Dharmadasa, Alicia Northall, Kevin Talbot, Alexander G Thompson, Martin R Turner

**Affiliations:** Nuffield Department of Clinical Neurosciences, University of Oxford, Oxford OX3 9DU, UK; Oxford Centre for Human Brain Activity, Wellcome Centre for Integrative Neuroimaging, University of Oxford, Oxford OX3 7JX, UK; Nuffield Department of Clinical Neurosciences, University of Oxford, Oxford OX3 9DU, UK; Oxford Centre for Human Brain Activity, Wellcome Centre for Integrative Neuroimaging, University of Oxford, Oxford OX3 7JX, UK; Nuffield Department of Clinical Neurosciences, University of Oxford, Oxford OX3 9DU, UK; Oxford Centre for Human Brain Activity, Wellcome Centre for Integrative Neuroimaging, University of Oxford, Oxford OX3 7JX, UK; Nuffield Department of Clinical Neurosciences, University of Oxford, Oxford OX3 9DU, UK; Nuffield Department of Clinical Neurosciences, University of Oxford, Oxford OX3 9DU, UK; Nuffield Department of Clinical Neurosciences, University of Oxford, Oxford OX3 9DU, UK; Oxford Centre for Human Brain Activity, Wellcome Centre for Integrative Neuroimaging, University of Oxford, Oxford OX3 7JX, UK

**Keywords:** MRI, DTI, fMRI, neuropathology, handedness

## Abstract

Amyotrophic lateral sclerosis (ALS) is a progressive neurodegenerative disease of the motor system marked by significant phenotypic heterogeneity. Motor symptoms in the limbs consistently emerge focally and asymmetrically and, whilst variable, the pattern of regional progression related to the balance of clinical upper and lower motor neuron signs, upper versus lower limb onset and hand dominance to some extent. The neurobiological mechanisms and pathological correlates for this lateralized onset and non-random progression are uncertain. Cerebral neuroimaging studies have commonly reported structural and functional asymmetries in ALS, but the limited analysis of the pre-symptomatic phase has limited their implications. Post-mortem study of spinal cord provided strong evidence for focal pathology at symptom onset in ALS. Histopathological staging of molecular pathology in post-mortem tissue lacks clinical correlation and an ordered, sequential temporal progression in life cannot be assumed. The development of integrated brain and cord MRI holds the hope of deepening understanding of the relationship between focal symptomatology and histopathological progression. This review considers the nature and implications of asymmetry in ALS across clinical, neuroimaging and post-mortem histopathology, highlighting the current gaps in knowledge and the need for a broader investigative framework.

## Teaser

Amyotrophic lateral sclerosis (ALS) is marked by significant phenotypic heterogeneity. By examining clinical, neuroimaging and post-mortem histopathological evidence, Yoganathan *et al*. consider whether the disease process in ALS is fundamentally asymmetric.

## Introduction

Amyotrophic lateral sclerosis (ALS) is a neurodegenerative syndrome characterized by the progressive disintegration of the motor system, encompassing its wider brain connections in a clinicopathological spectrum with frontotemporal dementia (FTD).^1,2^ ALS is characterized by the loss of upper motor neurons (UMNs) of the motor cortex and corticospinal tract (CST), and the lower motor neurons (LMNs) of the brainstem nuclei and spinal cord anterior horns. There is significant phenotypic heterogeneity in relation to the site of first symptoms, the relative balance of UMN and LMN signs, the extent of cognitive and behavioural involvement, and rate of clinical progression.^3^ Despite this clinical variation and strong evidence for multiple upstream biological pathways underpinning disease risk and progression, the vast majority of ALS cases are defined by a common histopathological signature of neuronal and glial cytoplasmic aggregates of phosphorylated transactive response DNA binding protein (TDP-43).^4,5^

Focal onset of weakness is almost universal in ALS. The insightful observations of Gowers in the late 19th century encapsulate the typical onset and progression of ALS symptoms: ‘from the part [of the limb] first affected the disease spreads to other parts of the same limb. Before it has attained a considerable degree in one limb, it usually shows itself in the corresponding limb on the other side; often in the muscles corresponding to those in which it commenced’.^6^

The timing of initial functional impairment is most consistently used to anchor the symptom onset in ALS. The location of initial loss-of-function in ALS is variable but not random. Across diverse populations, it is consistently reported in a limb (equally either arm or leg) in two-thirds of cases, and in the muscles of speech in approximately one-quarter, with a small minority consisting of initial paraspinal or diaphragm muscle weakness.^7^ Symptom onset is therefore consistently reported as very focal, often with a clear timing to a given month. For limb-onset ALS, this will usually be perceived as initially clearly unilateral.

The neurobiological underpinning of focal and lateralized onset of symptoms in ALS with apparent orderly spread is unclear and intersects with wider debates about whether the breakdown of corticomotorneuronal integrity first emerges within the spinal anterior horns (or even more distally) and spreads rostrally (termed ‘dying back’), or if it is initiated within the brain and moves caudally (‘dying forward’). Whilst a pathological process that begins focally and spreads through the network is an appealing hypothesis, there are alternative explanations for lateralized focal onset, e.g. a generalized system failure superimposed on a structurally and functionally asymmetrical motor system. This review considers pathological asymmetry in the human motor system in health and in ALS.

## Observations of asymmetry in the motor system

### Physiological motor system asymmetry

The human nervous system contains a remarkable degree of differentiation, evolved over several million years through the selection pressure associated with increased specialization, notably for language and social cognition, upright posture for walking, and fine motor control for dexterity.^8^ Conspicuous features of motor system evolution include the emergence of monosynaptic connections within the corticomotorneuronal system associated with faster motor processing, and the development of cerebral dominance and laterality that is presumed to also offer functional efficiencies.^9^ Despite the superficially symmetrical anatomy of the human brain and spinal cord, the execution of human function is remarkably lateralized. This can be attributed to the asymmetrical distribution of grey matter (GM) and white matter (WM) in the left and right brain hemispheres, and associated differences in functional connectivity within the neural circuits of each hemisphere.^10^

Within the structure of the motor cortex, its thick GM layers reflect an intricate web of connections to the CST, the primary projection pathway governing voluntary movements. Fritsch and Hitzig’s discovery in 1870 that electrical stimulation of specific motor cortex regions could evoke regional muscle movements, paved the way for the concept of somatotopy subsequently represented as an ‘homunculus’, with lateralized brain function an inherent feature of the motor system.^11^ Comparative histological studies of the spinal cord across primate species revealed a marked increase in the proportion of WM occupied by corticospinal fibres in humans.^12^ Inspection of the human spinal cord has shown that 74% of spinal cords exhibit asymmetry, 73% of which are larger on the right side attributable to a disproportionate number of corticospinal fibres decussating from left to right, leading to a larger right lateral corticospinal tract. Correspondingly, the anterior corticospinal tract on the left side—where fibres have not crossed—is typically smaller. This asymmetry is not associated with handedness. The anterior corticospinal tract is primarily involved in controlling axial musculature, while the lateral tract has a predominant role in limb movement. The discrepancy in tract size suggests that the right side of the spinal cord may receive a larger influx of corticospinal fibres, which affects both axial and limb musculature, independent of the dominant hemisphere.^12^

### Insights from neuroimaging

Non-invasive neuroimaging, particularly MRI with enhanced resolution, has established the most accurate *in vivo* links between structural and functional brain asymmetries. Voxel-based morphometry (VBM) and allied tools, such as cortical thickness measurement, compare GM tissue between groups. Cortical asymmetries in brain structure include the left hemisphere exhibiting greater thickness in the anterior cortex, while rightward asymmetry is prevalent in the posterior cortex.^9,13^ Paired subcortical structures also display volumetric asymmetry, e.g. the thalamus, putamen and pallidum being larger in the left hemisphere, while the hippocampus, amygdala, nucleus accumbens and caudate nucleus are larger in the right hemisphere. Leftward asymmetry in global cortical thickness, and specifically in the putamen, also appear to increase with age,^14^ a factor that may hold relevance for neurodegenerative diseases that predominantly surface later in life.^9,14,15^

In addition to volume-based methods, more advanced structural and functional MRI approaches further demonstrate brain asymmetries. Diffusion tensor imaging (DTI) is an MRI-based technique used to characterize WM tracts, by using the orientational properties of water molecule diffusion to provide estimates of microstructural integrity, expressed as fractional anisotropy (FA).^16^ Functional MRI (fMRI) is based on the detection of synchronous regional patterns of spontaneous blood oxygen level-dependent (BOLD) signal fluctuation. This approach can provide a measure of functional connectivity (FC) when acquired in the task-free (so-called ‘resting’) state. DTI has revealed a broad leftward asymmetry consistent between measures of microstructure and connectivity in WM.^17^ While fMRI studies point to more extensive functional connectivity in the right hemisphere, the direction of this asymmetry appears to shift at various stages of development. Lateralized differences in connectivity have been observed, wherein the left hemisphere exhibits greater functional intra-connectivity, particularly associated with language and fine motor coordination, while the right hemisphere extends its interactions more across both hemispheres (greater functional inter-connectivity). This has been postulated to facilitate visuospatial and attention processing.^9,18^

### Hand dominance

The notion that lateralization of the human brain confers a functional benefit is underscored by fMRI studies linking the intensity of lateralization to cognitive proficiency.^18-21^ Such lateralization potentially facilitates concurrent task processing by the brain’s hemispheres. A pronounced functional human motor asymmetry is evident in handedness, manifesting early in development. As early as 15 weeks, the developing foetus has been detected to exhibit a right upper limb preference, moving and sucking the right thumbs more frequently than the left, a predilection that aligns with later handedness^22^ and likely involves genetic factors.^23^ If both parents are right-handed, their child has a 10% chance of being left-handed. However, with one or both left-handed parents, the likelihood increases to 22% and 27%, respectively.^24^ The prevalence of left- or mixed-handedness is strikingly increased from ∼10% in the general population^21^ to between 20% and 40% in schizophrenia,^25^ though it is not seen in ALS.^26,27^

The structure and morphology of the central sulcus (CS) at the level of the hand primary motor cortex, has also been shown to be related to hand preference and skill.^21^ For example, in right-handed males, the maximum finger tapping rate of the dominant (right) hand correlated positively with the contralateral (i.e. left) central sulcus GM volume, and negatively with the ipsilateral (i.e. right) central sulcus GM volume. However, in left-handed males, the maximum finger tapping rate of the non-dominant (right) hand was strongly correlated with the GM volume of the ipsilateral central sulcus but not significantly with that of the contralateral central sulcus.^28^ Taken together, these results suggest that hand function and GM volume of the corresponding hand region are more tightly coupled for the dominant hand. In addition, MRI has shown that the depth of the central sulcus is also related to handedness.^29^ In right-handers, the left central sulcus is deeper than the right, and vice versa in left-handers. The macrostructural asymmetry in right-handers is complemented by a microstructural left-larger-than-right asymmetry in neuropil volume in Brodmann’s area (BA) 4. In addition, microstructural MRI has highlighted that age-related iron accumulation is minimal in the upper limb region of M1 compared to the lower limb and bulbar regions, specifically in the dominant hemisphere.^30^ These asymmetries suggest that hand preference is associated with distinctive microstructural properties underpinning differences in the organization of motor systems between left and right-handers.

Functional disparities associated with handedness have also been noted, particularly in motor cortex engagement. In a study using transcranial magnetic stimulation (TMS) on the motor cortex of left- and right-handed individuals, motor evoked potentials remained consistent across both sides. However, the dominant hand exhibited a shorter silent period duration, attributed primarily to the withdrawal of corticospinal input to spinal motor neurons. The increased engagement of the CST during non-dominant hand contractions suggests an asymmetry in cortical inhibitory functions.^31^

Although fMRI of the primary motor cortex (M1) often displays unilateral activations during simple finger movements tasks, ipsilateral M1 activations are frequently observed, particularly with more complex or sequential tasks.^21^ In right-handers, the left M1 is activated during left and right-hand movement, whereas the right M1 is activated predominantly with left hand movement, implying functional asymmetry between left and right motor cortices.^32^ Moreover, left-handed subjects appear more symmetric in terms of M1 functional activation, with the right and left motor cortices activating similarly during ipsilateral hand moves.^32^ The cause of these ipsilateral activations remains unclear. A study combining repetitive TMS and fMRI demonstrated that right-handed subjects showing activation of the left motor cortex during ipsilateral hand moves were characterized by a stronger inhibition from the right versus left M1, indicating that ipsilateral activation was linked to reciprocal transcallosal inhibition, possibly to suppress mirror movements.^33^ Overall, ipsilateral activations raise the interesting possibility that anatomy of both contra- and ipsilateral central sulcus may be relevant to unilateral hand skill.^28^

In summary, the human motor system is remarkably lateralized, despite its superficially symmetrical anatomy. A multitude of neuroimaging studies have illustrated that hand dominance correlates with various structural, functional and morphological asymmetries of the central sulcus. While these hemispheric distinctions in a healthy brain are now better understood, their implications for neurological disorders remain unclear, specifically, whether the inherent lateralized nature of the brain’s motor system could hold clues to the typically asymmetrical onset of ALS.

## Observations of asymmetry in ALS

**Figure 1:**
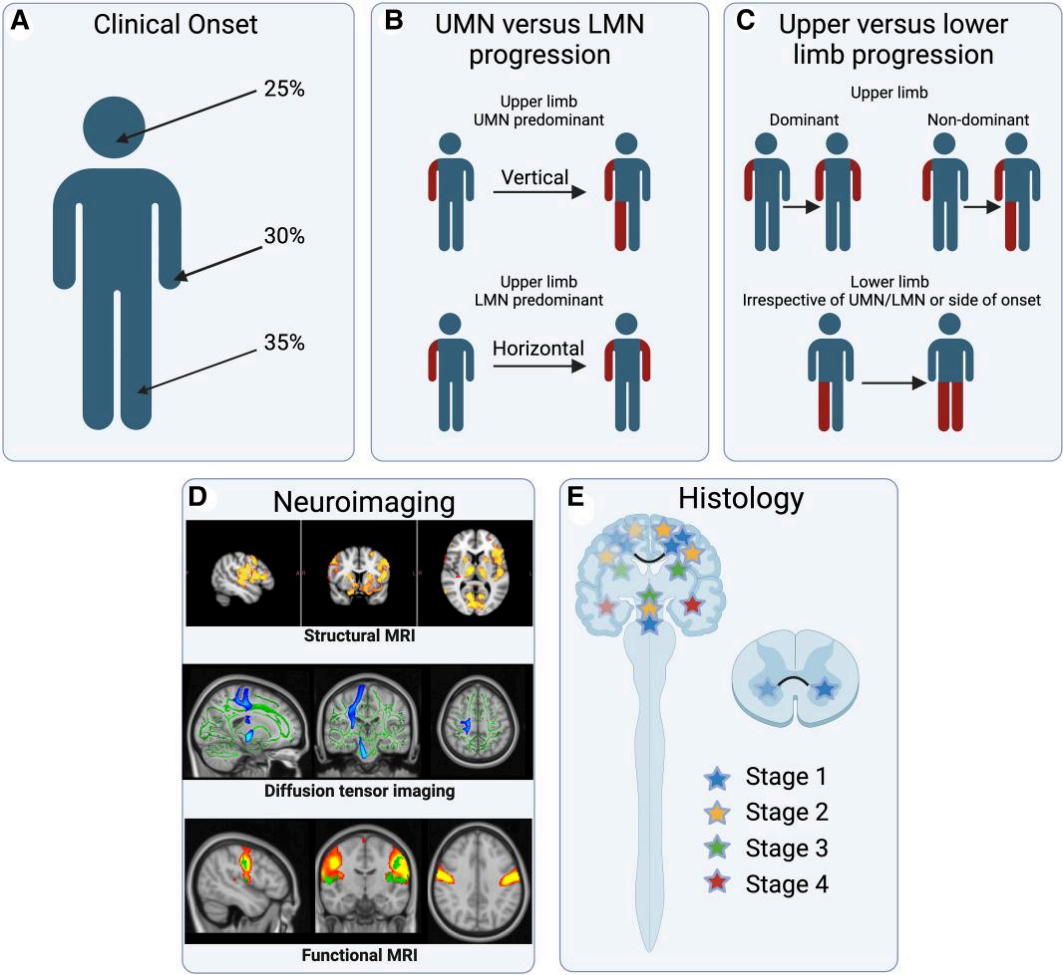
**Evidence sources for pathological asymmetry in ALS.** In the approximately two-thirds of cases for which the onset of symptoms is with limb weakness (**A**), this is strikingly focal and lateralized. Bulbar-onset of symptoms may be considered to be bilateral but this may simply be lack of clinicopathological resolution. The progression of symptoms is influenced by the predominance of upper motor neuron (UMN) versus lower motor neuron (LMN) clinical involvement (**B**), as well as by the dominant upper limb. However, this is less consistent in cases with lower limb symptom onset (**C**). Advanced structural MRI studies in amyotrophic lateral sclerosis (ALS) (**D**) have frequently reported lateralized volumetric and white matter tract involvement, but with very limited correlation to the anatomical site of initial weakness e.g. homuncular somatotopic representation. Functional MRI studies have tended to show more symmetrical activation, which might reflect compensatory recruitment of less affected brain regions. Post-mortem histopathological studies in the ALS cord have shown that pathology is maximal at the level and side of initial weakness (**E**, represented by darker stars, including the cord in the case of limb onset ALS). Within the cerebral histopathological Braak staging system, this key issue has not been considered systematically, due to the limitations of end-stage study, and so a stereotyped, sequential pattern of spread of ALS pathology during life cannot be assumed. Created in BioRender. Yoganathan, K. (2025) https://BioRender.com/u29y282.

Evidence sources for pathological asymmetry in ALS are summarized in Fig. 1.

### Search strategy and selection criteria

We performed a narrative systematic review of the relevant literature targeting adult populations, searching databases such as OVID, MEDLINE, PsycINFO, PubMed, EMBASE and the Cochrane Library. The literature search included articles in the English language published up to 1 January 2024, employing a combination of keywords including ‘ALS’, ‘amyotrophic lateral sclerosis’, ‘MND’, ‘motor neuron disease’, ‘neuroimaging’, ‘MRI’, ‘magnetic resonance imaging’, ‘DTI’, ‘diffusion tensor imaging’, ‘EEG’, ‘electroencephalogram’, ‘MEG’, ‘magnetoencephalography’, ‘MRS, ‘magnetic resonance spectroscopy’, ‘PET’, ‘positron emission tomography’, ‘neuropathology’ and ‘histology’.

**Figure 2:**
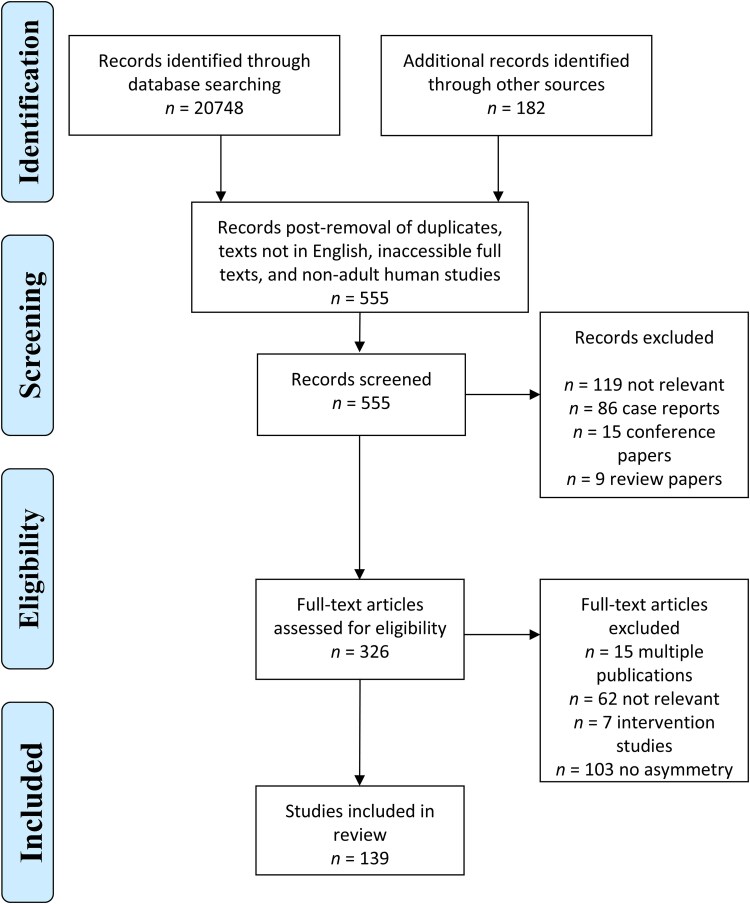
**PRISMA diagram of paper selection for inclusion in the systematic review**.

Articles were screened for relevance, with papers that met our criteria incorporated into the review. We also considered noteworthy papers identified within these reference lists. The assessment process for titles, abstracts and full texts was undertaken independently by two reviewers (K.Y. and A.N). In the event of discordant opinions, consensus was achieved through dialogue and a secondary examination. Inclusion criteria required patients diagnosed with ALS and a detailed description of the clinical, neuroimaging or neuropathological findings. We excluded conference abstracts, poster abstracts, non-peer-reviewed articles, editorials, letters, book chapters, book reviews, single case reports and review papers. Relevant papers were selected for full paper analysis as per the Preferred Reporting Items for Systematic Reviews and Meta-Analyses (PRISMA) guidelines (Fig. 2). Our search strategy was devised in adherence to the PRISMA guidelines.^117^ Due to the heterogeneity across studies, a quantitative meta-analysis was not viable, so a narrative synthesis was performed. This study is registered with PROSPERO ID CRD42023461521.

For details of the search strategy, see Table 1, Fig. 2 and Supplementary material.

**Table 1:** Electronic search strategy

Search line	Search term^a^	Results per line
1	[(ALS or amyotrophic lateral sclerosis or MND or motor neuron disease) and (neuroimaging or MRI or magnetic resonance imaging or DTI or diffusion tensor imaging or MEG or magnetoencephalography or EEG or electroencephalogram or PET or Positron emission tomography or MRS or magnetic resonance spectroscopy or neuropathology or histology)].mp. [mp = ti, ab, hw, tn, ot, dm, mf, dv, kf, fx, dq, bt, nm, ox, px, rx, ui, sy, ux, mx, tc, id, tm]	20 748
2	Limit 1 to ‘all adult (19 plus years)’	17 648
3	Limit 2 to adulthood <18+ years>	16 853
4	Limit 3 to (adult <18 to 64 years> or aged <65+ years>)	8330
5	Limit 4 to full text	1625
6	Limit 5 to ovid full text available	525
7	Limit 6 to english language	522
8	Limit 7 to human	520
9	Remove duplicates from 8	373
10	Additional records from references	182
Total results after de-duplication		555

^a^Databases included: Ovid MEDLINE(R) ALL (1946 to present); Embase (1974 to present); PsycINFO (1806 to present); Cochrane Central Register of Controlled Trials (CENTRAL); and Cochrane Database of Systematic Reviews (CDSR).

### Clinical asymmetry

In an ALS clinicopathological cohort, 98% of limb-onset motor manifestations were reported as focal and lateralized.^34^ The pattern of weakness also appeared more asymmetrical in upper limb- versus lower limb-onset ALS.^35^ In the upper limbs, a strong right-sided and hand-dominant predominance of initial symptoms has been reported (64–70% of cases occurring in the dominant upper limb), whereas in the legs, no predominant side of onset is observed.^26,36^

The progression of initial muscle weakness in ALS is not indiscriminate,^37^ with clinical evidence indicating that handedness and clinical phenotype shape the direction of progression. In a study of 138 patients, those who experienced initial weakness in a non-dominant limb were significantly more likely to develop weakness in the other limb on the same side, compared to those with initial weakness in a dominant limb (53% versus 23%, *P* = 0.008).^36^ Additionally, the relative distribution of upper limb UMN signs was affected by whether weakness first occurred on the dominant or non-dominant side, with those initially affected in the non-dominant limb having a greater asymmetry of UMN signs.^36^ In contrast, the pattern of spread in lower limb-onset ALS reported that initial progression was more often to the contralateral leg (76%) than the ipsilateral arm (24%), irrespective of the side of onset; similarly the distribution of UMN signs was symmetrical regardless of side of onset in lower limb onset cases. The initial side of onset was not a dependent factor for progression, although the time for progression to another limb was a major determinant of overall survival.^37^ Taken together, these findings suggest that handedness influences symptom onset and progression differently for patients with upper and lower limb-onset ALS.

Clinical UMN and LMN manifestations after early focal motor symptoms have been reported to peak in the same peripheral body region, before extending into neighbouring areas.^7,36,38,39^ The balance between UMN and LMN involvement may influence whether the initial spread is ipsilateral or contralateral.^36,40,41^ In a study of 913 patients, 89% showed focal versus multifocal-generalized spreading.^40^ Eight hundred and sixty-four individuals with asymmetrical upper or lower limb-onset tended to show ‘horizontal’ (contralateral) symptom progression (77%). ‘Vertical’ (ipsilateral) spread (21%) was linked to higher UMN burden and shorter survival. Proximal limb symptoms were more frequently associated with symmetrical onset, while distal symptoms were more frequently associated with asymmetric onset.^40^ Additionally, one study that analysed the characteristic patterns of muscle weakness across three independent patient cohorts (total 436 patients) demonstrated that the distribution of muscle weakness mirrored the patterns of corticomotorneuronal connections. This reinforces the idea that disease spread in ALS may follow the anatomical organization of motor networks.^42,43^

Although some studies analysed ALS subgroups with isolated upper or lower motor neuron involvement, such as primary lateral sclerosis (PLS), progressive muscular atrophy (PMA), or hereditary motor neuropathies (e.g. SOD1 mutations), the majority lack sufficient data for meaningful comparisons. Future large-scale studies focusing on these specific subgroups may provide deeper insights into the role of motor neuron predominance in disease asymmetry and progression. However, such studies would require careful stratification and longitudinal follow-up to account for variability and potential overlap in clinical presentations over time.

In summary, clinical observations indicate that symptoms of ALS typically begin in one area of the body and spread to adjacent regions, with the direction of spread being influenced by the balance of UMN and LMN signs and whether symptoms were first experienced in upper or lower limb. Hand dominance influences the side of symptom onset, progression to other limbs and relative asymmetry of UMN and LMN signs but only in upper limb onset ALS.

### Neuroimaging asymmetry

Despite advances in the understanding of *in vivo* pathology afforded by advanced MRI, it has been hard to demonstrate a simple relationship between anatomical measures and disability-based clinical correlations in ALS.^44^ There is some evidence from MRI studies that the initial limb of onset is correlated to appropriate somatotopic focal GM atrophy in the motor homunculus.^45^ A VBM study revealed right-handed ALS subjects with dominant limb symptom onset disproportionately lost GM in the left (contralateral) upper limb region, but this was not demonstrable for the lower limb cortical representation area,^46^ as noted in the limb dominance-symptom onset concordance study.^45^ ALS patients with a dominant limb onset to their weakness also showed unequal atrophy of areas involved in language and communication (left superior and transverse temporal gyri). This shift potentially illustrates the interplay of language dysfunction within the ALS-FTD spectrum. Comparing ALS-only patients with ALS-FTD patients revealed predominantly left frontal regions were significantly more atrophied in the ALS-FTD group, especially left middle and inferior frontal gyri.^47^ It seems clear that the brain is inherently more lateralized for language functions, and speech may be interconnected within a unified communication network, intertwining gestures and vocalizations.^48,49^ Such considerations may help understand the shared network vulnerability in the ALS-FTD spectrum.^50^

As white matter tracts degenerate in ALS, MRI-based diffusion becomes less directional and so the metric FA decreases, often with an increase in the more global metric of mean diffusivity (MD).^51-55^ Studies have revealed consistent changes in the CST and corpus callosum,^16,56^ but a variety of other non-motor tracts and networks may also be affected,^57^ with frontal lobe connections linked to cognitive dysfunction.^58^

In studies separating the CST into precentral and postcentral parts and with fibre-tracking analysis, the precentral CST mean FA differed significantly bilaterally in ALS versus healthy controls. In the postcentral CSTs however, the reduction of FA in the ALS patients, while highly significant, was only noted on the right.^59,60^ The study participants were mostly bilaterally affected, with no correlation of DTI findings to the initial side of symptom onset, despite one study revealing a broader correlation between CST FA and global disability score.^59-61^

Degeneration of the corpus callosum is a consistent finding in ALS, extending rostrally and bilaterally to the region of the primary motor cortex, independent of the degree of UMN involvement.^56^ Subregional variations in corpus callosum integrity in ALS were examined in relation to the presence of unilateral or bilateral limb weakness. The most prominent differences in diffusivity metrics were in the rostral body, posterior midbody and isthmus of the corpus callosum and loss of integrity was most prominent in the subgroup with unilateral limb weakness at the time of scanning.^62^ This group had a higher clinical UMN burden score (mean 12.6 versus 9.6), which independently correlated with the degree of corpus callosum involvement. Consequently, conducting longitudinal studies at regular intervals in patients with unilateral limb weakness, prior to the onset of bilateral weakness, could prove useful for further assessment.

Asymmetrical differences in FA in ALS patients compared to healthy controls have also been reported in the thalamus, pons, cingulate, uncinate fasciculus, insula, premotor cortex, supplementary motor area (SMA) and parietal lobe regions.^63-70^ Iron-sensitive MRI has revealed increased iron levels in cortical motor regions corresponding topographically to the initial site of ALS symptoms both in relation to side and anatomical site (upper limb, lower limb or bulbar).^71,72^ Despite small sample sizes, the high resolution afforded by 7 T MRI has localized this effect to the deep cortical layers and has shown that this occurs before significant atrophy.^73^

The clinical heterogeneity of the study cohorts, variation in control populations, scanners and aspects of analysis (e.g. significance thresholding) makes confidence in a pathobiological mechanism underpinning lateralized differences at this structural level very limited.

Resting state fMRI studies in ALS have yielded varied results for similar reasons.^74^ Increased FC in ALS patients, particularly within primary sensorimotor, premotor and anterior cingulate, showed a pronounced left-sided bias, especially evident in those with faster disease progression.^75^ Seed-based analysis shows reduced FC between right and left motor cortices in patients with limb-onset ALS.^76^ Consistent, apparently asymmetric, findings in ALS patients include regional FC alterations in the left M1, the left primary somatosensory cortex and the right SMA.^76-78^ Recent work suggests that advanced ALS is characterized by a transition from increased to decreased global functional connectivity,^79^ which may lend clarification to the mixed previous findings.

In task-based fMRI studies, ALS patients demonstrated extended ipsilateral activation^80^ and extended activation in the contralateral sensorimotor cortex during a motor task,^81^ as well as decreased regional activation during motor imagery.^82,83^ In addition, ALS patients showed reduced activation in the primary somatosensory and frontal dorsal areas and extended activation in premotor and frontoparietal motor regions during motor tasks, which matched with cortical atrophy.^84,85^ Beyond motor regions, ALS patients showed decreased regional activity in the right hemisphere during emotion processing tasks.^86^

The inherent reserve capacity of biological systems results in the emergence of symptoms in neurodegenerative disorders occurring late in the pathological cascade, and exploration of the early underpinnings of selective vulnerability is challenging for conditions like ALS, that are not reliably predictable from apparently healthy states. However, one study employing Mendelian randomization looking at premorbid brain structures, found that increased WM volume in the cerebral hemispheres were causally associated with ALS, and weaker causal associations were noted in the brainstem GM volume, parieto-occipital WM volume and left thalamic ventral anterior nucleus.^87^ In addition, those forms of ALS associated with highly penetrant monogenetic variants have provided a window on pre-symptomatic events. Imaging studies reveal that pre-symptomatic *C9orf72* mutation carriers show compromised WM integrity, especially in the left corpus callosum and cingulum, and disrupted connectivity in the sensorimotor and default mode networks, and most notably in the salience network and thalamus-seeded networks.^88^ These carriers also present with thinner cortical regions and smaller left caudate and putamen, unlike *SOD1* carriers, who do not exhibit these subcortical changes,^89,90^ but exhibit reduced FA changes in the posterior limb of internal capsule.^91^ Overall, this suggests that while ALS symptoms typically emerge after considerable pathological progression, genetic predispositions can offer early insights into disease pathogenesis.

### Histopathological asymmetry

Post-mortem study of ALS patient spinal cords has provided the most compelling evidence for focal pathology in relation to symptom onset. Early research indicated that motor neurons are systematically organized in the spinal cord’s anterior horns, with each cluster controlling a specific muscle and adjacent clusters serving functionally related muscles.^92^ One early study in ALS patients revealed a variable decrease in motor neuron numbers, with the extent of loss differing widely between individuals. This study did not consistently show a column-like organization of motor neurons in the human spinal cord. They did, however, observe considerable asymmetry in neuron loss, both within individual spinal levels and across different regions of the spinal cord.^93^ In a later study, 14 out of 19 cases showed regional anterior horn cell loss was graded radially away from the region of onset. In two further cases, LMN loss was minimal, coinciding with patients whose motor manifestations had been predominantly UMN.^94^ UMN and LMN degeneration was shown to be maximal at the level and side of the innervated body region that first manifested motor deficits, but with a dichotomy between them for relative severity of involvement and outward spread.^94^ A combination of rostral divergence to separate peripheral regions versus convergence upon the same region, differing in intensity and timing, was postulated.^34^ A broader, speculative hypothesis by the same author envisaged a contralateral corresponding somatotopic cortical focus of pathology, with possible transcallosal spread.^95^ This was aligned to a related corticomotorneuronal theory of ALS pathology and a corticofugal predominance to its origin and spread.^96,97^ The emergence of a clinicopathological overlap of ALS and FTD, and many neuroimaging studies revealing cerebral changes in asymptomatic carriers of monogenetic forms of both disorders (*C9orf72*-related reviewed in Li Hi Shing *et al.*^98^), has cemented this view. However, it is clear that there is significant presymptomatic spinal cord change,^99,100^ with machine-learning studies supporting a consistent primacy for this pathology.^101^ Furthermore, a cross-sectional MRI study comparing ipsilateral with contralateral limb spread of symptoms did not find corresponding callosal changes.^62^

A rare case of right hemiplegic ALS accompanied by aphasia provided more concrete evidence for a rostral to caudal spread of pathology.^102^ Post-mortem atrophy in the left hemisphere, accompanied by neuronal loss, gliosis and TDP-43-positive neuronal and glial cytoplasmic inclusions, was complemented by spinal cord examination. As expected, there was asymmetrical microglial infiltration predominantly in the crossed lateral corticospinal tract of the right cord, consistent with the hemiplegia observed. This finding aligns with the anatomy of the corticospinal tract, where the majority of motor fibres decussate at the pyramids in the medulla and descend contralaterally to innervate limb muscles. Additionally, microglial activation was observed in the uncrossed anterior corticospinal tract, which primarily descends ipsilaterally and is responsible for axial and proximal muscle control. The involvement of these specific tracts indicates that the degeneration is not limited to the dominant crossed fibres but extends systematically along both crossed and uncrossed pathways, consistent with the hierarchical anatomy of corticospinal projections. This selective pattern of degeneration, starting from the upper motor neurons in the cortex and propagating along descending motor pathways, provides concrete evidence for a rostral-to-caudal spread of pathology in ALS.

The study of the post-mortem distribution patterns of TDP-43 inclusions across 76 ALS brains parcellated these into four so-called ‘stages’.^103^ In those cases presenting with minimal TDP-43 pathology (Stage 1), the lesions were identified within the agranular motor cortex, brainstem motor nuclei of cranial nerves V, VII and X–XII, and spinal cord α-motor neurons. Cases with a greater burden of pathology showed involvement of the prefrontal neocortex (middle frontal gyrus), brainstem reticular formation, pre-cerebellar nuclei, and the red nucleus (Stage 2). Stage 3 was classified as TDP-43 pathology involving the prefrontal (gyrus rectus and orbital gyri), postcentral neocortex and striatum. Cases with the greatest burden showed TDP-43 inclusions in anteromedial portions of the temporal lobe, including the hippocampus (Stage 4). Importantly, as the authors state explicitly, there was no link to any clinical parameters, including disease duration, site of initial symptom onset or cognitive involvement, so that it is not possible to infer from this study that there is a stereotyped, ‘connectomic’ spread of pathology in ALS.

No hemispheric lateralization of pathology was noted in the above study. This stands in contrast to FTD-predominant syndromes, where post-mortem evaluations have consistently revealed characteristically asymmetric TDP-43 distribution, with a pronounced presence in the language-dominant hemisphere.^104-106^ While TDP-43 subtypes, such as type C, are frequently associated with semantic variant primary progressive aphasia (svPPA)—a subtype of FTD characterized by asymmetric temporal lobe degeneration—there is currently no specific information on TDP-43 subtypes in ALS studies, which limits our understanding of their potential contribution to asymmetry in ALS. Future research focusing on the classification of TDP-43 subtypes in ALS could provide valuable insights into shared or distinct pathological mechanisms within the ALS-FTD spectrum. Interestingly, in a post-mortem study of a single asymptomatic carrier of the *C9orf72* expansion,^107^ no pathological lateralization of the observed expansion-related dipeptide repeat pathology was noted.

## Conclusions

The clinical data from ALS patients provide compelling evidence for a focal initiation of symptoms, but evidence from a range of sources suggests that the emergence of weakness marks the end of long pre-clinical prodrome.^108,109^ The concordance of laterality of upper limb onset ALS and handedness is a potential clue to cerebral network-based influences, reflecting architectural properties of the motor system. For example, the size of Betz cells, characteristically depopulated in ALS,^110^ decreases along the mediolateral gradient of M1.^111^ There may be independent inhibitory interneuronal local circuit influences in relation to handedness^31^ and in ALS more widely.^112^ Broader hypotheses include premorbid cerebral neuronal networks as defining patterns of selective vulnerability and frequency of neurodegenerative disorders, including ALS.^113-116^

Neuroimaging studies have been largely focused on the brain until recently. Albeit *in vivo*, in those with symptomatic ALS, imaging has still been performed at a late stage and is striking in its lack of consistently asymmetrical or focal changes. This disparity might not only question the sensitivity of current imaging techniques but also suggest that the disease’s focal appearance could be the result of more complex, perhaps stochastic, processes within the motor network.

Histopathological evidence is—by its nature—end-stage, but the spinal cord evidence nonetheless strongly supports the concept of a focality of cord pathology in relation to the first symptoms. Furthermore, observational *in vivo* analysis corroborates the concept that contiguous spread of weakness is typical in ALS, perhaps through multiple mechanisms in which motor phenotypes characterized by a predominant UMN involvement are associated with an apparently ‘vertical’ ipsilateral pattern of disease progression, while those with predominant LMN involvement display ‘horizontal’ spreading across the spinal cord.

While our review highlights the clinical, imaging and histopathological manifestations of asymmetry in ALS, the absence of a unifying mechanism explaining this asymmetry underscores the complexity of the disease and emphasizes the need for further research to explore potential genetic, developmental and environmental contributors to this phenomenon. The relative paucity of pathological and imaging evidence for brain asymmetry stands in contrast to the distinct clinical manifestations of focality. This discrepancy could indicate that the scope of current research methodologies might not sufficiently capture the breadth of ALS’s complexity or point towards the alternative conceptualization of ALS as a condition with a diffuse pathology that is network-driven rather than focal. This review serves as a synthesis of existing evidence on asymmetry in ALS, highlighting key patterns and knowledge gaps, rather than providing immediate clinical applications. It is intended to serve as a foundation for future research into the pathophysiology and clinical implications of asymmetry in ALS.

In conclusion, a focal, and so inherently lateralized, pathology in ALS cannot be inferred from the currently published data. Temporal primacy of cortical processes has not been established with certainty and the possibility of a multi*-*focal onset to ALS remains an important possibility to pursue. This might involve more anatomically discrete networks of neurons, in which visible spinal cord pathology reflects more rostral events that are simply invisible to current tools. This necessitates a broader investigative framework, incorporating advanced imaging modalities and analytical techniques capable of capturing the disease’s progressive nature. Specifically, integrated studies combining brain and spinal cord MRI, particularly in patients with monomelic onset, could offer new insights into the interplay between focal symptomatology and the underlying disease process.

## Supplementary Material

awaf121_Supplementary_Data
